# Generalized vesicular rash in a child: a case of chickenpox (Varicella)

**DOI:** 10.11604/pamj.2025.52.169.49768

**Published:** 2025-12-18

**Authors:** Shardul Timande, Bibin Kurian

**Affiliations:** 1 Department of Child Health Nursing, Smt Radhikabai Meghe Memorial College of Nursing, Datta Meghe Institute of Higher Education and Research, Wardha, Maharashtra, India

**Keywords:** Chickenpox, varicella, healing without scar, vesicles and pustules, conservative management

## Image in medicine

A 10-year-old male child presented with a generalized vesicular rash for 4 days, associated with fever, itching, and malaise. There was no history of recent vaccination or contact with a known case of varicella. On examination, the child had multiple lesions in various stages of evolution - macules, papules, vesicles, pustules, and crusts - distributed over the face, trunk, and extremities, with a characteristic “dew drop on rose petal” appearance. No neurological or respiratory complications were noted. The patient was managed conservatively with antipyretics, calamine lotion, oral antihistamines, and adequate hydration. Acyclovir was not indicated, as the illness was mild and the child was immunocompetent. The lesions healed with no scarring after 10 days.

**Figure 1:**
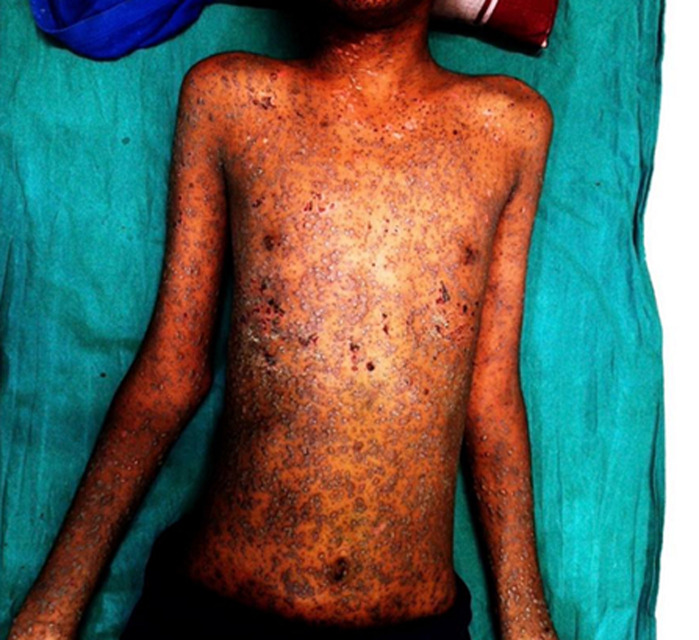
generalized vesicular rash in a child

